# Previous schistosome infection and the risk of incident hyperuricemia: A prospective cohort study

**DOI:** 10.1371/journal.pntd.0014608

**Published:** 2026-08-10

**Authors:** Congyue Wang, Zijian Tian, Ying Yang, Yushan Yue, Kaixin Zhou, Chong Li, Ke Lu

**Affiliations:** 1 Nosocomial Infection Management Department, Kunshan First People’s Hospital, Suzhou, Jiangsu, China; 2 Kunshan Biomedical Big Data Innovation Application Laboratory, Suzhou, Jiangsu, China; 3 National Laboratory of Biomacromolecules, Institute of Biophysics Chinese Academy of Sciences, Beijing, China; 4 Department of Rehabilitation, Kunshan First People’s Hospital, Suzhou, Jiangsu, China; 5 Guangzhou National Laboratory, Guangzhou International Bio Island, Guangzhou, Guangdong, China; 6 Department of Orthopedics, Traditional Chinese Medicine Hospital of Kunshan, Suzhou, Jiangsu, China; 7 Department of Orthopedics, Kunshan First People’s Hospital, Suzhou, Jiangsu, China; George Washington University School of Medicine and Health Sciences, UNITED STATES OF AMERICA

## Abstract

**Introduction:**

Although prior research in China has linked previous schistosome infection (PSI) to lower risks of metabolic syndrome and cardiorenovascular conditions, the association between PSI and hyperuricemia, a recognized risk factor for these conditions, remains unclear. This study examines the relationship between PSI and hyperuricemia and identifies factors associated with hyperuricemia incidence in individuals with PSI.

**Methods:**

This prospective cohort study included 23,051 adults from eight health centers in Kunshan (2018–2021). Exposure was defined by self-reported PSI, validated by liver B-ultrasound. The primary outcome was hyperuricemia. Baseline and follow-up data encompassed height, weight, blood pressure, serum uric acid, glucose, renal function parameters, liver function, and lipid profiles. Cox proportional hazard models were employed to analyze the relationship between PSI and hyperuricemia, adjusting for demographic, lifestyle, and clinical variables.

**Results:**

Among the 23,051 participants included in this study, 4,677 (20.29%) had a history of schistosome infection, and 87.53% of those with PSI were over the age of 64. During 60,335 person-years of follow-up, 4,417 incident cases of hyperuricemia were recorded. The incidence rate was 64.91 per 1,000 person-years in the PSI group and 75.38 per 1,000 person-years in the non-PSI group. The overall cumulative hazard differed significantly among individuals, with the incidence of hyperuricemia being 17.36% (95% CI: 16.28–18.48%) and 19.62% (95% CI: 19.05–20.20%) in PSI and Non-PSI groups. After adjusting for confounders, PSI was associated with an 11% lower risk of hyperuricemia (HR: 0.89, 95% CI: 0.82–0.97, *p* = 0.005).

**Conclusions:**

In this prospective cohort study, PSI was observed to be associated with a lower risk of incident hyperuricemia. This finding reflects an observational association and does not establish causality. From a public health standpoint, schistosome infection remains a serious disease requiring continued prevention and control. Further investigations are needed to clarify the underlying mechanisms linking PSI to hyperuricemia.

## Author summary

Our research explores a surprising connection between a serious parasitic disease, schistosomiasis, and a common metabolic disorder, hyperuricemia. While we know schistosomiasis is harmful, our analysis of human health data revealed an unexpected pattern: there appears to be a link between the infection and a lower risk of developing high uric acid levels. We believe this might be due to the way the parasite alters the body’s internal environment, potentially dampening the inflammatory responses that drive uric acid production. This finding is important because it adds to the growing evidence that our evolutionary history with parasites might have shaped our immune systems in ways that affect modern diseases. However, we stress that this does not mean the infection is beneficial. Schistosomiasis causes significant suffering and must be prevented. By understanding this complex biological interaction, we hope to uncover new clues about how to manage metabolic diseases without the risks of actual infection.

## Introduction

Hyperuricemia is a chronic disorder characterized by elevated serum uric acid and is associated with a diverse array of diseases, including metabolic, cardiovascular, and kidney-related conditions [1]. Hyperuricemia serves as the principal risk factor for gout and can give rise to kidney stones as well as being the most prevalent form of inflammatory arthritis [2,3]. Prevalence rates include 13.3% in China, 21.4% in the U.S., and 25.8% in Japan [4–6]. Furthermore, asymptomatic hyperuricemia independently increases the risk of cardiovascular diseases [7–9].

Schistosomiasis, a neglected tropical disease caused by *Schistosoma* blood flukes, remains a major global public health concern, particularly in regions such as the Middle East, South America, Southeast Asia, and sub-Saharan Africa [10,11]. Affecting an estimated 240 million people across 78 countries, with 800 million at risk, the disease imposes a substantial health burden [12]. Annually, schistosomiasis accounts for 1.532 million disability-adjusted life years (DALYs) lost, 77% of which occur in sub-Saharan Africa [13–16].

The core endemic areas for schistosomiasis in China are now largely concentrated along the middle and lower Yangtze River, accounting for over 92% of reported national cases [17,18]. Kunshan City, the study site, is located in the lower reaches of the Yangtze River in Jiangsu Province. Geographically situated within the water-network endemic region of the Yangtze River delta (31°06′–31°32′N, 120°48′–121°09′E), Kunshan historically faced significant schistosomiasis transmission risks. Beyond being a parasitic infection, schistosomiasis also manifests as an immunological disorder [19,20]. Experimental studies in animals suggest that schistosome antigens can significantly alter metabolic profiles while triggering strong anti-inflammatory responses [21,22].

The clinical features and outcomes of acute and chronic *Schistosoma japonicum* infections are well-documented, but the long-term health impacts of previous schistosome infection (PSI) remain poorly understood. Recent epidemiological studies conducted in China have reported inverse associations between PSI and several components of the metabolic syndrome, including diabetes, obesity, and dyslipidemia [22–25]. Chronic schistosome infection induces a persistent shift from Th1 to Th2 dominated responses and expansion of regulatory T cell networks [19,20], while hyperuricemia is increasingly recognized as a disorder driven by low-grade sterile inflammation via urate crystal induced NLRP3 inflammasome activation [7–9]. We hypothesized that this anti-inflammatory milieu might attenuate the inflammatory cascades contributing to urate dysregulation, resulting in a lower incidence of hyperuricemia among individuals with PSI. To test this hypothesis, we conducted a prospective cohort study in Kunshan City.

## Methods

### Ethics statement

The study protocol was established, according to the ethical guidelines of the Helsinki Declaration and was approved by the ethics committee of a class A tertiary hospital of Kunshan. Informed written consent of all participants has been obtained (2023-03-014-H01-K01-A01).

## Study design and population

The Kunshan Cohort, established between 2014 and 2023, includes 50,000 participants recruited from eight community health centers in Kunshan. This cohort is affiliated with a local Class A tertiary hospital, a partner university, the Kunshan Health and Family Planning Information Center, as well as primary hospitals and community health centers. The data were extracted from electronic health records (EHR) at community health centers. These records combined information from annual government-subsidized examinations for residents aged 65 and above, along with routine healthcare visits from adults aged 18–64 [26–28]. Informed consent was obtained electronically using an iPad-based system. Participants provided consent via electronic signature or fingerprint identification, in accordance with ethical guidelines. Ethical approval for the study was granted by the local hospital’s research ethics committee, and written informed consent was obtained from all participants.

To address limitations arising from the small sample size between 2014 and 2017 and the lack of long-term follow-up for participants in the Kunshan cohort from 2022 to 2023, an initial group of 36,011 participants from the 2018–2021 Kunshan cohort was considered. However, individuals with pre-existing hyperuricemia at baseline (n = 6,672), missing baseline (n = 2,717) or follow-up (n = 2,470) uric acid data, incomplete lifestyle and biochemical information (n = 454), or missing duplicate GLOBAL_INDEX values (a unique anonymized identifier used for data linkage in the local EHR system) (n = 647) were excluded from the analysis. Consequently, the final cohort included 23,051 participants (S1 Fig).

## Assessment and definitions of variables

Demographic and lifestyle variables, including marital status (married/unmarried/divorced/widowed), smoking status (current/former/never), drinking status (current/former/never), and outdoor walking frequency, were collected through standardized face-to-face questionnaires administered by trained staff. Resting blood pressure was measured twice on the right forearm using a standard mercury sphygmomanometer, with at least a five-minute rest period between measurements, conducted by a trained nurse or physician. Laboratory tests were performed to obtain fasting serum uric acid, fasting plasma glucose, renal function markers, liver function indicators, and lipid profile components.

PSI was assessed through self-reports validated by liver B-ultrasound. Hypertension was defined as SBP ≥ 140 mmHg or DBP ≥ 90 mmHg, and diabetes as FPG ≥ 7.0 mmol/L. Dyslipidemia criteria included TC ≥ 5.2 mmol/L, TG ≥ 1.7 mmol/L, HDL-C < 1.0 mmol/L, or LDL-C ≥ 3.4 mmol/L [29]. BMI classifications for Chinese individuals were: underweight(<18.5), normal (18.5–23.9), overweight (24.0–27.9), and obese (≥ 28.0) [30].

## Outcome ascertainment

Hyperuricemia was defined by serum uric acid levels exceeding 420 µmol/L (7.0 mg/dL) in men or 360 µmol/L (6.0 mg/dL) in women [31]. Newly diagnosed cases without prior history were classified as incident hyperuricemia.

## Statistical analysis

Categorical variables were presented as proportions, and continuous variables as medians with interquartile ranges (IQRs). Group comparisons used chi-square, Fisher’s exact, or Kruskal–Wallis tests. Follow-up duration was the time from recruitment to loss to follow-up, death, or December 31, 2021. Kaplan–Meier plots and log-rank tests compared cumulative hazards, while Cox regression models provided hazard ratios (HRs) with 95% confidence intervals (CIs). The proportional hazards assumption was assessed using Schoenfeld residuals. Model adjustments included significant univariable factors (Model 1), demographic and lifestyle variables (Model 2), comorbidities (Model 3), and laboratory markers (Model 4). Statistical significance was set at *p* < 0.05, and analyses were performed using R version 4.1.3.

Additional analyses examined PSI and hyperuricemia events across subgroups defined by demographic, lifestyle, and clinical factors.

## Results

### Baseline characteristics

Among the 23,051 participants with PSI included in this study, 10,400 (45.1%) were male, and the median age was 66 years ([IQR]: 61–71) (Table 1). Of these participants, 4,677 (20.29%) had a history of schistosome infection, and 87.53% of those with PSI were over the age of 64. Individuals with PSI were more likely to have a lower BMI, smaller waist circumference, lower education levels, and a greater tendency to smoke or drink. They were also less likely to be female or married. Furthermore, at baseline assessment, individuals with PSI showed a lower prevalence of diabetes and dyslipidemia. Other characteristics that differed significantly between the PSI and non-PSI groups included WBC, platelet count, total bilirubin (TBIL), aspartate aminotransferase (AST), alanine aminotransferase (ALT), blood urea nitrogen (BUN), hemoglobin levels, serum creatinine (SCR), and serum uric acid levels (SUA).

**Table 1:** Characteristics of the study population with and without PSI.

	Overall	Non-PSI	PSI	*p*
Characteristics	23051	18374 (79.71)	4677 (20.29)	
Age, years	66 (61–71)	65 (57–70)	70 (66–75)	<0.001
18 ~ 64 years	9059 (39.30)	8476 (46.13)	583 (12.47)	<0.001
≥ 65 years	13992 (60.70)	9898 (53.87)	4094 (87.53)	
Sex				
Male	10400 (45.12)	8026 (43.68)	2374 (50.76)	<0.001
Female	12651 (54.88)	10348 (56.32)	2303 (49.24)	
Education attainment				
No or others	4984 (21.62)	3670 (19.97)	1314 (28.09)	<0.001
Primary School	9528 (41.33)	7012 (38.16)	2516 (53.80)	
Secondary School	8539 (37.04)	7692 (41.86)	847 (18.11)	
Marital status				
No	2052 (8.90)	1505 (8.19)	547 (11.70)	<0.001
Yes	20999 (91.10)	16869 (91.81)	4130 (88.30)	
Smoking status				
No	17750 (77.00)	14301 (77.83)	3449 (73.74)	<0.001
Yes	5301 (23.00)	4073 (22.17)	1228 (26.26)	
Drinking status				
No	18767 (81.42)	15027 (81.78)	3740 (79.97)	0.005
Yes	4284 (18.58)	3347 (18.22)	937 (20.03)	
Outdoor walking status				
No	11764 (51.03)	9097 (49.51)	2667 (57.02)	<0.001
Yes	11287 (48.97)	9277 (50.49)	2010 (42.98)	
Waist, cm	84 (78–90)	84 (78–90)	81 (76–86)	<0.001
SBP, mmHg	137 (126–150)	137 (126–150)	138 (127–151)	<0.001
DBP, mmHg	81 (75–89)	82 (75–89)	80 (73–87)	<0.001
Hypertension				
No	11317 (49.10)	9026 (49.12)	2291 (48.98)	0.878
Yes	11734 (50.90)	9348 (50.88)	2386 (51.02)	
BMI, kg/m^2^	24.24 (22.22–26.49)	24.46 (22.5–26.7)	23.38 (21.46–25.43)	<0.001
Underweight	543 (2.36)	363 (1.98)	180 (3.85)	<0.001
Normal	10156 (44.05)	7587 (41.29)	2569 (55.43)	
Overweight	9294 (40.32)	7671 (41.75)	1623 (34.70)	
Obese	3058 (13.27)	2753 (14.98)	305 (6.52)	
FPG, mmol/L	5.6 (5.2–6.3)	5.6 (5.2–6.3)	5.6 (5.2–6.1)	<0.001
Diabetes				
No	19456 (84.40)	15335 (83.46)	4121 (88.11)	<0.001
Yes	3595 (15.60)	3039 (16.54)	556 (11.89)	
TG, mmol/L	1.3 (0.9–1.8)	1.3 (1–1.9)	1.1 (0.8–1.4)	<0.001
< 1.7 mmol/L	15984 (69.34)	12083 (65.76)	3901 (83.41)	<0.001
≥ 1.7 mmol/L	7067 (30.66)	6291 (34.24)	776 (16.59)	
TC, mmol/L	4.6 (4–5.2)	4.6 (4.1–5.3)	4.5 (4–5.1)	<0.001
< 5.2 mmol/L	16611 (72.06)	13067 (71.12)	3544 (75.78)	<0.001
≥ 5.2 mmol/L	6440 (27.94)	5307 (28.88)	1133 (24.22)	
HDL-C, mmol/L	1.4 (1.2–1.6)	1.4 (1.1–1.6)	1.5 (1.2–1.7)	<0.001
≥ 1.0 mmol/L	21316 (92.47)	16836 (91.63)	4480 (95.79)	<0.001
< 1.0 mmol/L	1735 (7.53)	1538 (8.37)	197 (4.21)	
LDL-C, mmol/L	2.5 (2–3.1)	2.6 (2–3.1)	2.5 (2–3)	<0.001
< 3.4 mmol/L	19598 (85.02)	15528 (84.51)	4070 (87.02)	<0.001
≥ 3.4 mmol/L	3453 (14.98)	2846 (15.49)	607 (12.98)	
Dyslipidemia				
No	11836 (51.35)	8851 (48.17)	2985 (63.82)	<0.001
Yes	11215 (48.65)	9523 (51.83)	1692 (36.18)	
WBC, 10^9^/L	5.8 (4.9–6.8)	5.8 (4.93–6.88)	5.5 (4.7–6.5)	<0.001
Platelet, 10^9^/L	193 (157–231)	197 (161–235)	178 (144–214)	<0.001
TBIL, µmol/L	10.8 (8.1–14.3)	10.7 (8–14.1)	11.2 (8.3–14.9)	<0.001
AST, U/L	20 (17–24)	20 (17–24)	20 (17–25)	<0.001
ALT, U/L	17 (13–23)	18 (14–23)	17 (13–22)	<0.001
BUN, mmol/L	5.5 (4.6–6.5)	5.5 (4.6–6.4)	5.8 (4.9–6.8)	<0.001
Hemoglobin, g/L	137 (128–147)	137 (129–147.75)	136 (127–145)	<0.001
SCR, µmol/L	69.3 (60–80)	69 (60–80)	71 (62–82)	<0.001
SUA, µmol/L	298 (254–339.25)	299 (255–340)	294 (250–337)	<0.001

Data are shown as IQR, no. (%) or no./total no. (%). *P* values were derived from chi-square test or Kruskal–Wallis test, as appropriate. Abbreviations: IQR, interquartile range; SBP, systolic blood pressure, DBP, diastolic blood pressure, BMI, body mass index, FPG, fasting plasma glucose, TG, plasma triglyceride level, TC, total cholesterol, HDL-C, high-density lipoprotein cholesterol, LDL-C, low-density lipoprotein cholesterol, WBC, white blood cell, TBIL, total bilirubin, AST aspartate aminotransferase, ALT alanine aminotransferase, BUN blood urea nitrogen, SCR serum creatinine, SUA serum uric acid.

## Associations between PSI and incident hyperuricemia

**Fig 1:**
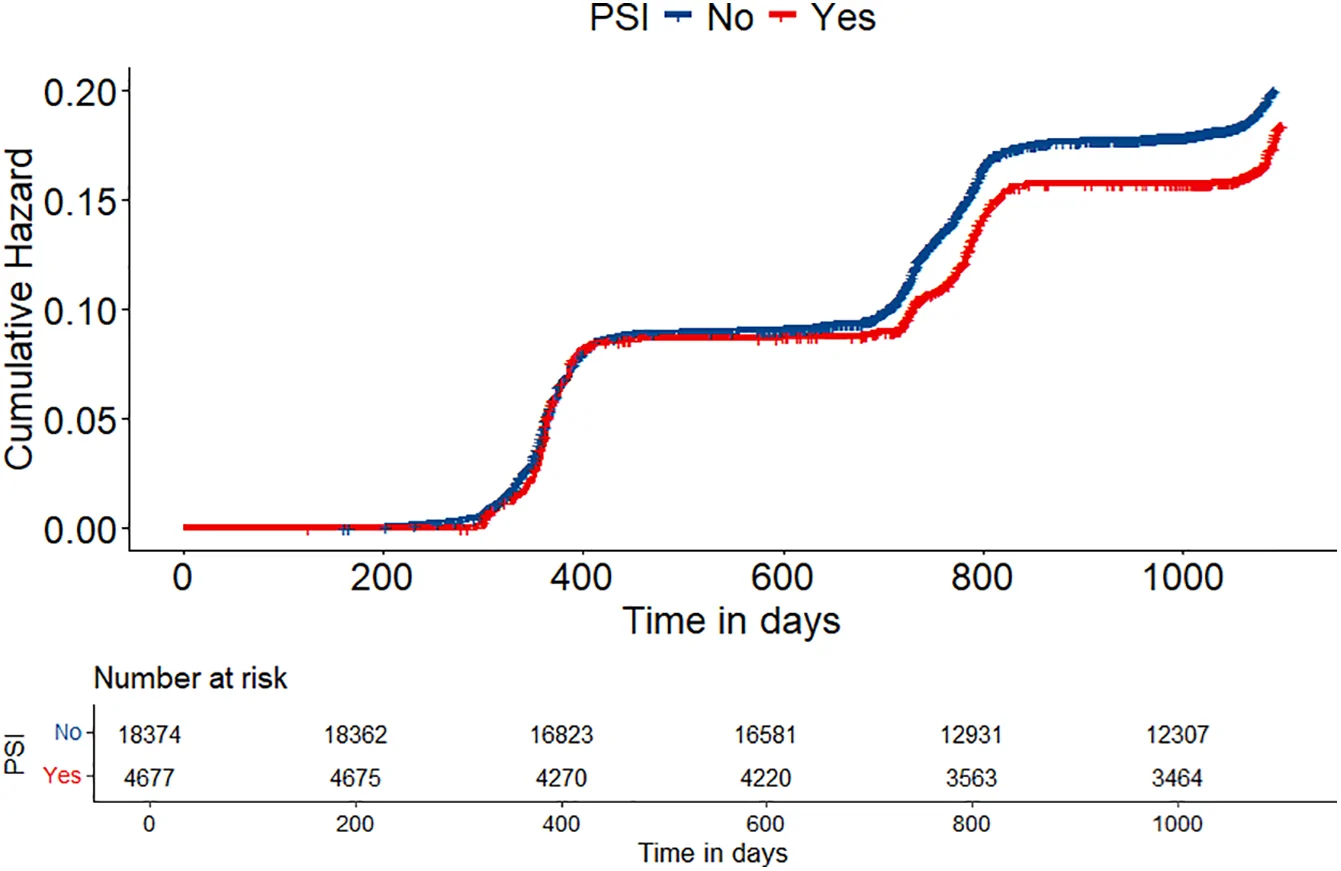
Cumulative hazard of incident hyperuricemia by PSI. The y-axis represents the cumulative hazard of hyperuricemia. Abbreviations: PSI, previous schistosomiasis infection..

After three years of follow-up, 4,417 cases of hyperuricemia were recorded over a total of 60,335 person-years. The incidence rate was 64.91 per 1,000 person-years in the PSI group and 75.38 per 1,000 person-years in the non-PSI group, yielding an incidence rate ratio of 0.86. The overall cumulative hazard varied significantly among individuals, with the incidence of hyperuricemia being 17.36% (95% confidence interval [CI]: 16.28–18.48%) in the PSI group and 19.62% (95% CI: 19.05–20.20%) in the non-PSI group (Fig 1) (*p* < 0.001). Significant differences were also observed when individuals were categorized based on predetermined covariates including age, marital status, BMI, drinking status, hypertension, and dyslipidemia; however, no significant differences were found for sex, smoking status, outdoor walking status, or diabetes (S2 Fig).

**Fig 2:**
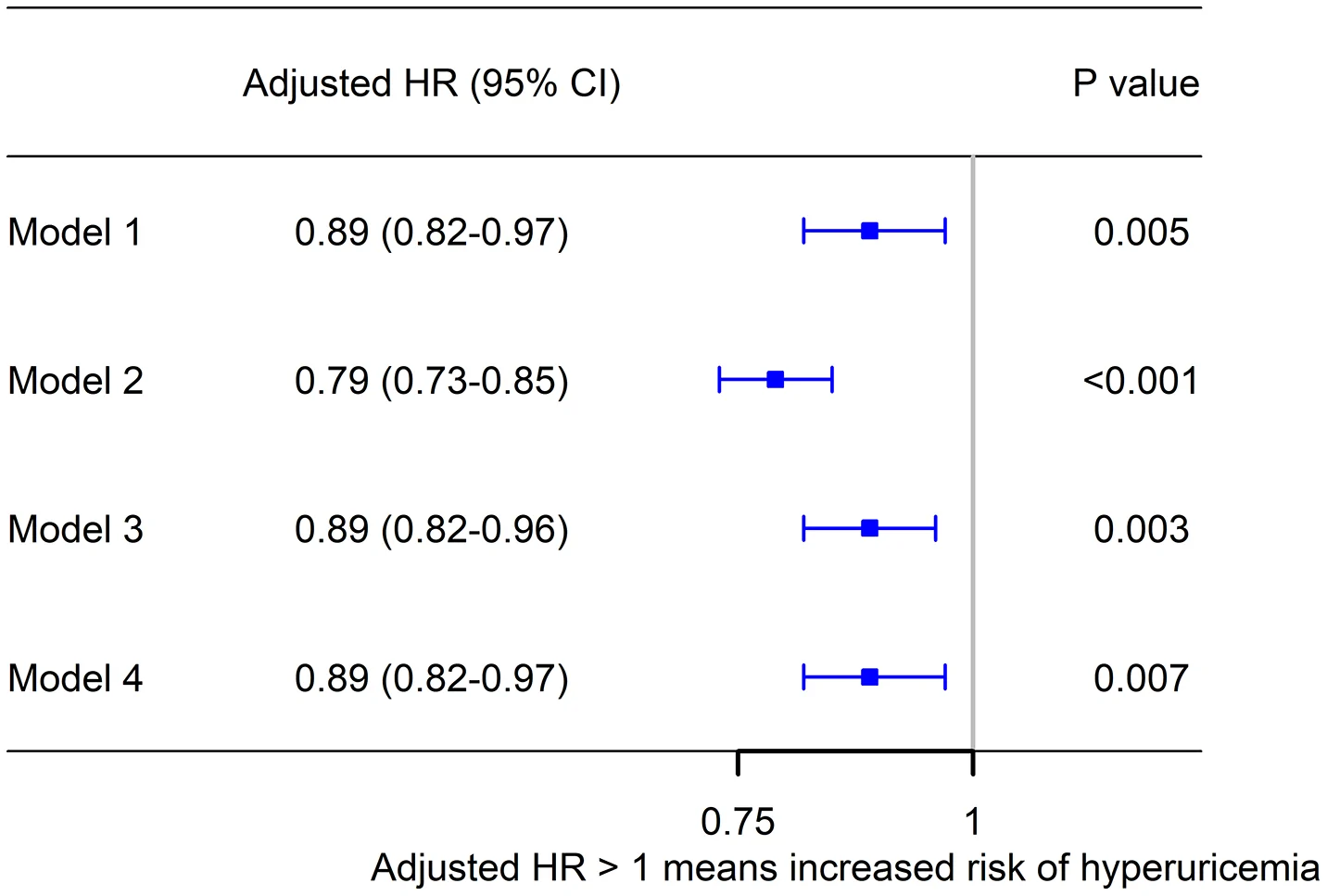
Multivariable analyses for the association between PSI and incident hyperuricemia. Model 1 was adjusted for significant univariable factors. Model 2 was adjusted for predefined confounding factors, including sex, age, marital status, educational attainment, smoking status, drinking status, and outdoor walking status. Model 3 further incorporated hypertension, dyslipidemia, diabetes, and BMI. Model 4 included the variables from Model 3 along with SCR, WBC, AST, ALT, BUN, and hemoglobin levels. Abbreviations: BMI body mass index, SCR serum creatinine, WBC white blood cell, AST aspartate aminotransferase, ALT alanine aminotransferase, BUN blood urea nitrogen. Underweight and Normal weight individuals were combined due to the small sample size of the underweight group.

In univariable analysis, PSI was linked to a 0.85-fold reduction in hyperuricemia risk (95% CI: 0.79–0.92, *p* < 0.001, Table 2). Among the covariates analyzed, age, marital status, drinking status, outdoor walking status, hypertension, BMI, and dyslipidemia were significantly associated with incident hyperuricemia. Among the continuous variables examined, WBC, AST, ALT, BUN, and SCR were also linked to incident hyperuricemia (Table 2). In multivariable analysis, PSI was linked to a 0.89-fold reduction in hyperuricemia risk (95% CI: 0.82–0.97, *p* = 0.005, Table 2, Fig 2 Model 1), with the HR unchanged after adjusting for all variables including demographic, lifestyle variables, comorbidities and laboratory markers (Fig 2). Schoenfeld residual tests confirmed that the proportional hazards assumption was not violated for the primary exposure variable (PSI, *p* = 0.121) or for the global model (*p* = 0.205).

**Table 2:** Univariable and multivariable analysis of risk factors for incident hyperuricemia.

Variable	No. of Events/All	Univariable		Multivariable	
		HR (95% CI)	*p*	Adjusted HR (95% CI)	*p*
Age,years					
18 ~ 64	1672/9059	Ref		Ref	
≥ 65	2745/13992	1.19 (1.12–1.27)	<0.001	1.27 (1.19–1.35)	<0.001
Sex					
Male	2010/10400	Ref			
Female	2407/12651	0.96 (0.91–1.02)	0.210		
Education attainment					
No or Others	974/4984	Ref			
Primary school	1784/9528	0.97 (0.9–1.05)	0.488		
Secondary school	1659/8539	0.98 (0.9–1.06)	0.588		
Marital status					
No	430/2052	Ref		Ref	
Yes	3987/20999	0.86 (0.78–0.95)	0.003	0.83 (0.76–0.92)	<0.001
Smoking status					
No	3408/17750	Ref			
Yes	1009/5301	1.01 (0.94–1.08)	0.836		
Drinking status					
No	3471/18767	Ref		Ref	
Yes	946/4284	1.22 (1.13–1.31)	<0.001	1.16 (1.08–1.25)	<0.001
Outdoor walking status					
No	2199/11764	Ref			
Yes	2218/11287	1.07 (1.01–1.13)	0.030	1.04 (0.98–1.11)	0.148
Hypertension					
No	1857/11317	Ref		Ref	
Yes	2560/11734	1.33 (1.25–1.41)	<0.001	1.20 (1.13–1.27)	<0.001
BMI, kg/m^2^					
Underweight and Normal	1510/10699	Ref		Ref	
Overweight	2047/9294	1.59 (1.49–1.7)	<0.001	1.47 (1.38–1.58)	<0.001
Obese	860/3058	2.08 (1.92–2.27)	<0.001	1.85 (1.69–2.02)	<0.001
Diabetes					
No	3723/19456	Ref			
Yes	694/3595	0.99 (0.92–1.08)	0.855		
Dyslipidemia					
No	1937/11836	Ref		Ref	
Yes	2480/11215	1.38 (1.3–1.47)	<0.001	1.28 (1.21–1.36)	<0.001
PSI					
No	3605/18374	Ref		Ref	
Yes	812/4677	0.85 (0.79–0.92)	<0.001	0.89 (0.82–0.97)	0.005
WBC, 10^9^/L		1.10 (1.08–1.12)	<0.001	1.06 (1.04–1.08)	<0.001
Platelet, 10^9^/L		1.00 (1.00–1.00)	0.586		
TBIL, µmol/L		1.00 (1.00–1.01)	0.152		
AST, U/L		1.00 (1.00–1.01)	<0.001	1.00 (1.00–1.01)	0.269
ALT, U/L		1.01 (1.00–1.01)	<0.001	1.00 (1.00–1.01)	0.141
BUN, mmol/L		1.03 (1.02–1.04)	<0.001	1.01 (1.00–1.03)	0.150
Hemoglobin, g/L		1.00 (1.00–1.00)	0.598		
SCR, µmol/L		1.00 (1.00–1.00)	<0.001	1.00 (1.00–1.00)	<0.001

Abbreviations: BMI, body mass index, TG, plasma triglyceride level, TC, total cholesterol, HDL-C, high-density lipoprotein cholesterol, LDL-C, low-density lipoprotein cholesterol, WBC, white blood cell, TBIL, total bilirubin, AST, aspartate aminotransferase, ALT, alanine aminotransferase, BUN, blood urea nitrogen, SCR, serum creatinine, SUA, serum uric acid. Underweight and Normal weight individuals were combined due to the small sample size of the underweight group.

**Fig 3:**
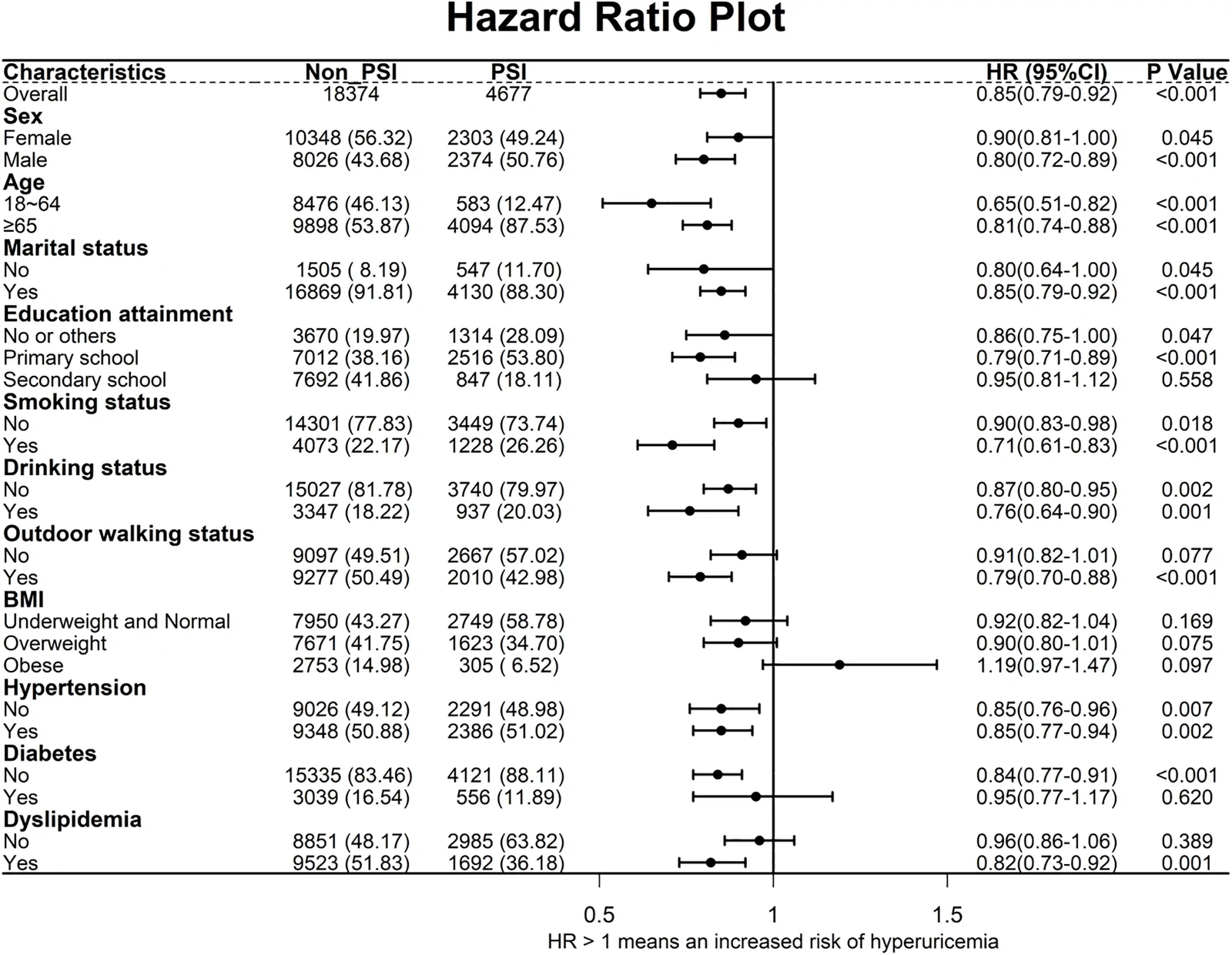
Subgroup analysis of the association between PSI and incident hyperuricemia. Abbreviations: PSI previous schistosomiasis infection, BMI body mass index.

In the subgroup analysis, decreased risks of incident hyperuricemia associated with PSI were observed across almost all subgroups; however, the HR appeared to be lower in male participants, younger individuals, and those who smoke or drink. Conversely, for participants with higher educational attainment or no outdoor walking status, the association between PSI and incident hyperuricemia was not statistically significant. Additionally, individuals with diabetes or without dyslipidemia did not show statistically significant results (Fig 3). Furthermore, in the subgroup stratified by BMI, the association between PSI and hyperuricemia was also found to be non-significant.

## Discussion

This study analyzed 23,051 Kunshan participants to assess the link between PSI and hyperuricemia risk. After adjustment, PSI was associated with an 11% to 21% lower risk of hyperuricemia. A small 2-year study in Suzhou found PSI reduced hyperuricemia risk, but its sample size and follow-up were limited [32]. We conducted a larger study to explore this relationship further. While the clinical mechanisms underlying the relationship between schistosomal infection and hyperuricemia remain incompletely understood, several studies have indicated that schistosomal infection may potentially influence urate metabolism through modulating immune responses, inflammatory status, and urate metabolism. Schistosome infection actively remodels T-cell responses with the aim of facilitating the Th1-Th2 shift, actuating regulatory cell networks, and eliciting a state of T-cell hyperreactivity [33–35]. Consistent with the notion that regulatory responses become more pronounced during chronic infection [36,37], this study found evidence of enhanced immunoregulation in the later stages of infection [38]. This immunoregulatory milieu may subsequently modulate host metabolic homeostasis, thereby influencing uric acid metabolism and inflammatory responses. Uric acid acts as an antioxidant, reducing oxidative stress [39–44], but elevated levels can turn pro-oxidant, exacerbating inflammation and impairing metabolic and vascular function [45–47].

The risk of hyperuricemia is influenced by genetic factors, environmental conditions, and individual traits like age, sex, and BMI [48,49]. Factors such as diet, alcohol use, obesity, insulin resistance, and certain medical conditions contribute to its development [50–53]. Our findings confirm that higher BMI, age, alcohol consumption, hypertension, dyslipidemia, and elevated WBC counts increase risk, while marriage lowers susceptibility.

Our Cox analysis showed that age and alcohol consumption are significantly linked to hyperuricemia risk. Participants aged 65 or older had a 1.27-fold increased risk, consistent with studies on aging and reduced uric acid clearance [54,55]. Alcohol consumption was associated with a 1.16-fold increased risk, boosting uric acid production and decreasing renal excretion [56,57].

The strong link between hypertension and hyperuricemia was also observed in our study, with hypertensive patients having an HR of 1.20 (95% CI 1.13-1.27) for hyperuricemia. This finding is supported by numerous studies that have shown a bidirectional relationship between hypertension and hyperuricemia, with each condition exacerbating the other and contributing to cardiovascular risk [58,59]. Additionally, the link between obesity and hyperuricemia was confirmed, with overweight individuals having an HR of 1.47, and obese individuals having an even higher HR of 1.85. These results suggest that obesity may contribute to hyperuricemia through increased insulin resistance and reduced uric acid excretion [60,61].

Our study also found a significant association between dyslipidemia and hyperuricemia risk, with an HR of 1.28. This finding is in line with previous research results, suggesting that dyslipidemia may promote the development of hyperuricemia by affecting uric acid excretion and increasing the inflammatory state [62,63].

In this study, although genetic factors were not included in the final predictive model, a systematic literature review revealed that genetic background plays a complex role in the occurrence and development of hyperuricemia. Previous genome-wide association studies have confirmed that gene loci such as SLC2A9, SLC22A12 and ABCG2 are significantly associated with uric acid metabolism, and these genes mainly participate in the renal tubular uric acid transport and intestinal excretion processes [1,64]. These findings align with prior studies indicating that the effect of a healthy lifestyle on hyperuricemia is less significant in those with higher genetic risk [65]. However, the polymorphism distribution of related single-nucleotide polymorphisms (SNP) loci in the study cohort did not show statistical significance, which may be related to the genetic heterogeneity of the study population, sample size limitations, or the interaction of environmental factors.

### Study advantages and limitations

The study is characterized by several key strengths. Its forward-looking design, substantial sample size, long-term follow-up, and robust data from the Kunshan cohort contribute significantly to its reliability. These elements allow for the inclusion of numerous potential confounders and ensure strong statistical power. Additionally, the participant pool primarily consisted of individuals with hyperuricemia, which ensured a uniform group.

Despite the strengths of this study, some limitations warrant consideration. First, although we accounted for various confounders and conducted several analyses, the observational design still leaves room for unmeasured confounding and potential bias. Additionally, the observed inverse association may be partly attributable to residual socioeconomic confounding. Individuals with PSI in this cohort were predominantly older rural residents. Limited access to purine-rich foods and alcohol, both well-established dietary drivers of hyperuricemia, may have lowered serum urate levels through dietary restriction in this population. Chronic hepatic impairment from prior infection may further reduce nutritional intake. These considerations underscore that this association should not be interpreted as a causal protective effect of PSI against hyperuricemia. Furthermore, the study’s participants were exclusively from the Kunshan region and of Han Chinese descent, which calls for further research that includes other regions and ethnic groups. Finally, while the findings of this population-based study strongly suggest that PSI has an observational association with hyperuricemia, definitive causal relationships cannot be established.

## Conclusion

This study found an inverse association between PSI and incident hyperuricemia. After adjusting for confounders, PSI remained significantly associated with lower hyperuricemia risk. However, this observational finding does not imply causation. While the link is noteworthy, it is crucial to emphasize that PSI remains a serious infectious disease requiring strict prevention. Further research is needed to clarify the underlying mechanisms of this association.

## Supporting information

S1 Fig Flow chart. (TIF)

S2 Fig Kaplan-Meier curves. (TIF)

## Abbreviation:

PSI: previous schistosome infection
DALYs: disability-adjusted life years
EHR: electronic health records
IQRs: interquartile ranges
HRs: hazard ratios
CIs: confidence intervals
SBP: systolic blood pressure
DBP: diastolic blood pressure
FPG: fasting plasma glucose
TBIL: total bilirubin
AST: aspartate aminotransferase
ALT: alanine aminotransferase
BUN: blood urea nitrogen
SCR: serum creatinine
SUA: serum uric acid levels
BMI: body mass index
TG: plasma triglyceride level
TC: total cholesterol
HDL-C: high-density lipoprotein cholesterol
LDL-C: low-density lipoprotein cholesterol
WBC: white blood cell
